# Whole genome microarray analysis of neural progenitor C17.2 cells during differentiation and validation of 30 neural mRNA biomarkers for estimation of developmental neurotoxicity

**DOI:** 10.1371/journal.pone.0190066

**Published:** 2017-12-20

**Authors:** Kristina Attoff, Anda Gliga, Jessica Lundqvist, Ulf Norinder, Anna Forsby

**Affiliations:** 1 Department of Neurochemistry, Stockholm University, Stockholm, Sweden; 2 Institute of Environmental Medicine, Karolinska Institutet, Stockholm, Sweden; 3 Swetox, Karolinska Institutet, Unit of Toxicology Sciences, Södertälje, Sweden; National Cancer Institute, UNITED STATES

## Abstract

Despite its high relevance, developmental neurotoxicity (DNT) is one of the least studied forms of toxicity. Current guidelines for DNT testing are based on *in vivo* testing and they require extensive resources. Transcriptomic approaches using relevant *in vitro* models have been suggested as a useful tool for identifying possible DNT-generating compounds. In this study, we performed whole genome microarray analysis on the murine progenitor cell line C17.2 following 5 and 10 days of differentiation. We identified 30 genes that are strongly associated with neural differentiation. The C17.2 cell line can be differentiated into a co-culture of both neurons and neuroglial cells, giving a more relevant picture of the brain than using neuronal cells alone. Among the most highly upregulated genes were genes involved in neurogenesis (CHRDL1), axonal guidance (BMP4), neuronal connectivity (PLXDC2), axonogenesis (RTN4R) and astrocyte differentiation (S100B). The 30 biomarkers were further validated by exposure to non-cytotoxic concentrations of two DNT-inducing compounds (valproic acid and methylmercury) and one neurotoxic chemical possessing a possible DNT activity (acrylamide). Twenty-eight of the 30 biomarkers were altered by at least one of the neurotoxic substances, proving the importance of these biomarkers during differentiation. These results suggest that gene expression profiling using a predefined set of biomarkers could be used as a sensitive tool for initial DNT screening of chemicals. Using a predefined set of mRNA biomarkers, instead of the whole genome, makes this model affordable and high-throughput. The use of such models could help speed up the initial screening of substances, possibly indicating alerts that need to be further studied in more sophisticated models.

## Introduction

During the last 3 decades, there has been an increase in the number of children diagnosed with learning and neurodevelopmental disorders. This alarming trend has given rise to an emerging need for good models and methods to evaluate possible developmental neurotoxicity (DNT) induced by exposure to different chemicals [1]. The current approved guidelines for toxicity testing rely solely on *in vivo* models using endpoints such as behavior, sexual maturation, brain weight and neuropathology, which are broad and unspecific. In some cases tests of neurobehavioral function (e.g., social behavior), neurochemistry or neuropathology (histological sections are examined microscopically to determine alterations) are also applied [2,3]. With regards to the vast quantity of chemicals being introduced to the market each year, *in vivo* DNT testing according to the existing guidelines is both time consuming, expensive, hard to interpret and comes with ethical costs [4]. According to the Toxicity testing in the 21^st^ century paradigm, there is a call for reliable *in vitro* methods that can provide rapid, high-throughput screening of chemicals [5]. However, due to the various uncertainties and problems mentioned for the current guidelines for DNT testing, there is also a great need of DNT screening specific alternative methods [6].

The central nervous system (CNS) is considered to be one of the most susceptible targets of systemic toxicity, and the developing nervous system is often even more sensitive [7]. DNT endpoints can be very challenging to study since the toxicity may not correlate with cell death but rather by subtle alterations in a number of specific and sensitive events that take place in an organized and controlled manner during development [8,9]. Since various parts and cell types of the brain develop during different time points, they are sensitive to toxic insults at different time windows [7]. All of these biological events during brain development, such as proliferation, migration, differentiation, apoptosis and synaptogenesis, can be targeted by xenobiotics and potentially lead to DNT [10]. Even subtle alterations of the ratio between different subpopulations of neural cells, the number of synapses, connectivity or the positioning of cells can give rise to DNT [11,12]. The next generation of DNT testing is envisioned to combine both *in silico* and *in vitro* testing methods in order to generate a more rapid and efficient toxicity screening [13]. For example, human embryonic stem cells have been shown to be a reliable tool for the identification of critical events during neural development [14]. Different endpoints such as neurite outgrowth [15,16] and neural proliferation [17] were reported to be important for detection of DNT. In addition, several studies identified biomarkers of neural differentiation that could be used for toxicity screening [12,14,18–22]. Hogberg et al identified the use of micro-electrode arrays in primary cultures of rat cortical neurons as an emerging technology to study DNT [23,24]. However, single endpoints/biomarkers will simply not suffice for *in vitro* DNT testing. Testing strategies for DNT should be comprehensive, *i*.*e*. include a battery of relevant endpoints and should give mechanistic insight that would provide information to discriminate between different neural subpopulations and different stages of neural differentiation. International stakeholders have proposed a DNT testing strategy based on compound testing across a battery of in vitro tests including the important factors of timing and processes of brain development [6]. The use of mRNA biomarkers is a good example of such an approach that has previously been reported for DNT test systems [21,25,26]. Expression monitoring by hybridization to high-density oligonucleotides is a sensitive, specific and quantitative method to monitor very large number of mRNAs [27,28].

The objective of the present study was to use transcriptomic microarray gene expression analysis in order to identify a panel of mRNA biomarkers that are critical for neural differentiation in the murine neural progenitor cell line C17.2. The C17.2 cell line is a multipotent progenitor cell line that upon differentiation with nerve growth factor (NGF) and brain derived neurotrophic factor (BDNF), can differentiate into a co-culture of neurons and neuroglial cells [29]. We identified 30 biomarkers that were validated using Reverse Transcription Real-Time Quantitative Polymerase Chain Reaction (RT-qPCR). In addition, we also evaluated the gene expression of the selected biomarkers following exposure to four xenobiotics. Two known DNT-inducing compounds were used as positive controls, *i*.*e*. methylmercury chloride (MeHg) [30] and valproic acid sodium salt (VPA) [31,32] and one neurotoxic and potentially DNT-inducing compound, *i*.*e*. acrylamide (ACR) [15,33]. D-mannitol was used as a negative control.

## Materials and methods

### Chemicals and reagents

Acrylamide (99.9% purity) (A9099), methylmercury chloride (442534), valproic acid sodium salt (P4543), D-mannitol (M1902), putrescine dihydrochloride (P5780), progesterone (P8783), sodium selenite (S5261), bovine insulin (I1882) were purchased from Sigma Aldrich (Sweden). Recombinant mouse β-nerve growth factor (NGF) (1156-NG) and recombinant human brain-derived neurotrophic factor (BDNF) (248-BD) were purchased from R&D systems (United Kingdom). Apo-transferrin from bovine plasma (02152334) was purchased from MP Biomedicals. GIBCO^®^ phosphate-buffered saline (PBS), GIBCO^®^ Trypsin/ EDTA (0.05/0.02%), GIBCO^®^ Dulbecco’s modified Eagles medium (DMEM), GIBCO^®^ Dulbecco’s modified Eagles medium: Nutrient mixture F-12 (DMEM/F-12), horse serum, fetal calf serum, GIBCO^®^ L-glutamine, GIBCO^®^ Pen-Strep (10,000 U/ml of penicillin and 10,000 μg/ml of streptomycin) and AlamarBlue^®^ cell viability reagent were purchased from ThermoFisher Scientific (Sweden). All plastics used for cell culturing were from Corning Inc., (Corning NY). RNA extraction kit RNeasy Plus Mini Kit was purchased from Qiagen. PrimePCR^™^ Positive Control SYBR^®^ Green Assay, PrimePCR^™^ DNA Contamination Control SYBR^®^ Green Assay, PrimePCR^™^ RNA Quality SYBR^®^ Green Assay, PrimePCR^™^ Reverse Transcription Control SYBR^®^ Green Assay, PrimePCR^™^ precasted 96-well plates, iScript cDNA synthesis kit and SsoAdvanced^™^ Universal SYBR^®^ Green Supermix for RT-qPCR were purchased from Bio-Rad (Sweden).

### Cell line and cell culturing

The neural progenitor cell line C17.2 was a generous gift from Professor Sandra Ceccatelli (Karolinska Institutet, Stockholm, Sweden) with permission of Professor Evan Snyder (Harvard Medical School, Boston, USA). The C17.2 cell line was originally cloned from mouse cerebellar neural progenitor cells, which were immortalized through *v-myc* retroviral transduction [34]. The cells were originally taken on postnatal day 4 from a male mouse. For routine cultures, the C17.2 cells were seeded at a density of 1.27 x 10^3^ cells/cm^2^ in cell culture Petri dishes. The cells were cultured in routine culture medium (DMEM supplemented with 5% horse serum, 10% fetal calf serum, 2 mM L-glutamine, 100 U penicillin/mL and 100 μg streptomycin/mL). The confluent cells were detached every 3,5 days using 0.05/0.02% trypsin/EDTA and seeded in a new cell culture Petri dish at the original density. For differentiation studies, the C17.2 cells were seeded in routine culture medium (see individual experiment for density). Twenty-four hours after seeding, the medium was changed to differentiation medium (DMEM/F-12 medium supplemented with 1 mM L-glutamine, 100 U penicillin/mL, 100 μg streptomycin/mL, modified N2 supplements (to a final concentration of 5 μg/mL bovine insulin, 20 nM progesterone, 30 nM sodium selenite, 100 μg/mL bovine apo-transferrin, and 100 μM putrescine dihydrochloride), 10 ng/mL NGF and 10 ng/mL BDNF). The differentiation medium was changed every 3^rd^ day for the duration of the differentiation. The cells were kept in a humidified atmosphere of 5% CO_2_ in air at 37°C.

### Culturing of cells for microarray expression analysis and RT-qPCR of selected biomarkers

For the experimental setup, the C17.2 cells were seeded in 10 cm diameter cell culture dishes in routine culture medium. The undifferentiated control cells were seeded at a density of 1.9 x 10^3^ cells/cm^2^ and the cells for differentiation were seeded at a density of 3.7 x 10^3^ cells/cm^2^. The control cells were seeded at a lower density than the cells seeded for differentiation due to the fact that they are known to spontaneously differentiate if the culture get too dense.

### mRNA extraction

After desired time of differentiation (5 or 10 days), the cells were harvested by trypsinization and cell pellets were collected. Undifferentiated cells were harvested after 3 days. The cell suspensions were centrifuged for 5 minutes at 500g and the pellet was stored at -80°C until mRNA extraction. On the day of the experiment, the cells were lysed and mRNA was extracted using the Qiagen RNeasy Plus Mini Kit according to manufacturer’s instructions. mRNA concentration was determined by a NanoPhotometer^™^ P-class (IMPLEN GmbH).

### Microarray expression analysis

The RNA quality was evaluated using the Agilent 2100 Bioanalyzer system (Agilent Technologies Inc, Palo Alto, CA). Two hundred and fifty ng of total RNA from each sample were used to generate amplified and biotinylated sense-strand cDNA from the entire expressed genome according to the GeneChip^®^ WT PLUS Reagent Kit User Manual (P/N 703174 Rev. 1, Affymetrix Inc., Santa Clara, CA). GeneChip^®^ ST Arrays (GeneChip^®^ Mouse Gene 2.1 ST 16-Array Plate) were hybridized for 16 hours in a 45°C incubator, washed and stained and finally scanned with the GeneTitan^®^ Multi-Channel (MC) Instrument, according to the GeneTitan Instrument User Guide for Expression Arrays Plates (P/N 702933 Rev. 2, Affymetrix Inc., Santa Clara, CA).

### Microarray data analysis

The raw data was normalized in the free software Expression Console provided by Affymetrix (http://www.affymetrix.com) using the robust multi-array average (RMA) method first suggested by Li and Wong in 2001 [35,36]. Subsequent analysis of the gene expression data was carried out in the freely available statistical computing language R (http://www.r-project.org) using packages available from the Bioconductor project (www.bioconductor.org). In order to search for the differentially expressed genes (DEGs) between the groups, an empirical Bayes moderated t-test was applied using the ‘limma’ package [37,38]. To address the problem with multiple testing, the p-values were adjusted using the method of Benjamini and Hochberg [39]. The microarray data have been deposited at Gene Expression Omnibus (GSE97337). Volcano plots were generated using R and the ggplot2 Bioconductor package. Venn diagrams of the DEGs for each treatment vs the untreated control at the same point were plotted with a web tool developed by the Bioinformatics & Evolutionary Genomics Laboratory at VIB/UGent, Belgium (http://bioinformatics.psb.ugent.be/webtools/Venn/). Genes with a false discovery rate (FDA) adjusted p-value < 0.05 and absolute log2(fold change) > 1 were considered as differentially expressed. The lists of genes for plotting the Venn diagrams were based on the analysis-ready genes (see below).

### Principal component analysis

The outcome from the experiments was analyzed with principal component analysis (PCA) using SIMCA v14.0 (MKS data analytics solutions). The differences between the 3 repeated experiments for undifferentiated cells and 5 or 10 days of differentiation, respectively, as well as the differences between 10 and 5 days of differentiation were used as input variables for the PCA, i.e. each sample of undifferentiated cells were compared to all 3 samples of cells differentiated for 5 or 10 days and so on. The data were mean-centered and auto-scaled to unit variance and 7-fold cross-validations were used to determine the number of significant PCA components.

### Downstream analysis

Downstream analysis of the DEGs was performed using Ingenuity Pathway Analysis software (IPA, content version 26127183, Ingenuity Systems, Redwood City, CA). The contrast Day 5 vs Day 0 (undifferentiated cells) had 1190 differentially expressed (absolute log2(fold change) > 1, p-value < 0.05) probe set IDs out of which 1108 were mapped to gene symbols (using IPA). After removing duplicates there were a total of 1065 analysis-ready genes (665 upregulated, 400 downregulated). The contrast Day 10 vs Day 0 had 2458 differentially expressed (absolute log2(fold change) > 1, p-value < 0.05) probe set IDs out of which 2232 were mapped to gene symbols (using IPA). After removing duplicates there were a total of 2166 analysis-ready genes (1216 upregulated, 950 downregulated). The contrast Day 10 vs Day 5 had 307 differentially expressed (absolute log2(fold change) > 1, p-value < 0.05) probe set IDs out of which 285 were mapped to gene symbols (using IPA). After removing duplicates there were a total of 283 genes ready for analysis (192 upregulated, 91 downregulated). The analysis-ready genes were used for canonical pathway analysis as well as disease and function analysis. Output data were used to generate heatmaps of the top 20 enriched pathways according to the p-value as well as z-score (measure of pathway activation/inhibition). To select relevant biomarkers for neural differentiation of the C17.2 cell line, gene set enrichment analysis (GSEA) was performed on the genes selected as differentially expressed (absolute log2(fold change) > 1, p-value < 0.05) and from the genes that were determined as differentially expressed, all genes involved in gene sets connected to the brain and neural functions were further selected. From this list of differentially expressed genes involved in neural differentiation, the 30 genes with the highest log2(fold change) changes were chosen without bias. These 30 genes were further validated by RT-qPCR (http://software.broadinstitute.org/gsea/index.jsp). For the selected 30 genes, we performed gene ontology (GO) enrichment analysis using the WebGestalt online tool [40].

### Reverse Transcription Real-Time Quantitative Polymerase Chain Reaction

The selected mRNA biomarkers from the microarray were validated by RT-qPCR. Pre-casted white PrimePCR^™^ plates were designed and purchased from Bio-Rad. Two μg of each RNA sample (same samples as were used for the microarray analysis) were reverse transcribed into cDNA using iScript cDNA Synthesis Kit from Bio-Rad. Real-time qPCR reactions were carried out as described by Bio-Rad in the PrimePCR^™^ instruction manual, including experimental control assays for reverse transcription (PrimePCR^™^ Reverse Transcription Control SYBR^®^ Green Assay), genomic DNA (PrimePCR^™^ DNA Contamination Control SYBR^®^ Green Assay), RNA quality (PrimePCR^™^ RNA Quality SYBR^®^ Green Assay) and PCR performance (PrimePCR^™^ Positive Control SYBR^®^ Green Assay). It was performed in a CFX96 Touch^™^ Real-Time PCR Detection System (Bio-Rad) using SsoAdvanced^™^ Universal SYBR^®^ Green Supermix. The data were analyzed using the Bio-Rad CFX manager 3.1 software system. The samples were normalized against 3 reference genes; TATA box binding protein (Tbp), heat shock protein 90ab (Hsp90ab1) and ribosomal protein, large P1 (Rplp1) which were carefully selected so as to be equally expressed during all stages of differentiation relative to the total amount of mRNA [41].

### Chemical exposure

The substances were dissolved in differentiation medium and sterile-filtered through a 0.2 μm filter and then diluted to different concentrations. The substances were added in different concentration ranges depending on the substance; ACR range 0.1 nM-1 mM, MeHg range 10 pM-100 μM, VPA range 1 nM-10 mM and D-mannitol range 10 μM-100 mM. Cell cultures exposed to differentiation medium without any of the 4 substances were used as control. For all experiments, the exposure to the substances started 24 hours after seeding, at the same time as the change to differentiation medium. The medium was then changed every 3^rd^ day and the substances were added at every change of medium throughout the duration of the experiment. Fresh medium without any substance was added to control cells. A fresh stock solution and dilution series for each of the substances was prepared right before addition to the cells at each time of exposure and medium change.

### Determination of IC10 for the different substances using the AlamarBlue^®^ assay

The amount of viable cells was determined using the AlamarBlue^®^ cell viability reagent. For all experiments, the cells were seeded in clear 96-well plates. C17.2 cells were seeded at a density of 3.75 x 10^3^ cells/cm^2^. The cells were exposed to the substances diluted in differentiation medium for 10 days with the substance-containing medium changed every 3^rd^ day as described above. After completed exposure, the AlamarBlue^®^ reagent was added according to the manufacturer’s instructions, incubated for 1 hour and the absorbance was read at 570 nm using 600 nm as a reference wavelength. The inhibitory concentration 10% (IC10) was determined from nonlinear regression to fit the data to the log(inhibitor) vs response(variable slope) curve using the Hill slope (slope factor), equation Y = Bottom + (Top-Bottom)/(1+10^((LogIC10-X)*HillSlope)) (GraphPad Prism 7.02).

### Exposure of substances for validation of selected biomarkers

To further validate that the biomarkers selected from the microarray expression analysis that were validated by RT-qPCR, the biomarkers were analyzed after addition to 3 different neurotoxic substances and one negative control substance. The cells were seeded in 6 cm diameter cell culture dishes at a density of 3.7 x 10^3^ cells/cm^2^ and exposed during differentiation to the IC10 estimated from the the AlamarBlue^®^ assay described above; 70 μM of ACR, 90 nM of MeHg and 100 μM of VPA. D-mannitol didn’t show any cytotoxicity for the concentrations used, and 1 mM was chosen for cellular exposure. After 10 days of differentiation and exposure to the substances, the cells were harvested by trypsinization and cell pellets were collected. Cells were centrifuged for 5 minutes at 500g and the pellet was stored at -80°C until mRNA extraction. mRNA extraction, cDNA synthesis and RT-qPCR were performed in the exact same manner as stated above during the previous sections.

### Morphological evaluation

The cells were photographed using a phase contrast microscope (Olympus). Microscopy images were captured at 150x magnification using a CCD camera (Olympus DP50).

### Statistical analyzes for PrimePCR

GraphPad Prism 7.02 was used for statistical analysis of the data. Results were analyzed using one- or two-way ANOVA followed by Dunnett’s multiple comparisons test, * p<0.05, ** p<0.01, *** p<0.001 as compared to control.

## Results

### Microarray analysis of differentiating C17.2 reveals robust time-dependent changes of gene expression

**Fig 1:**
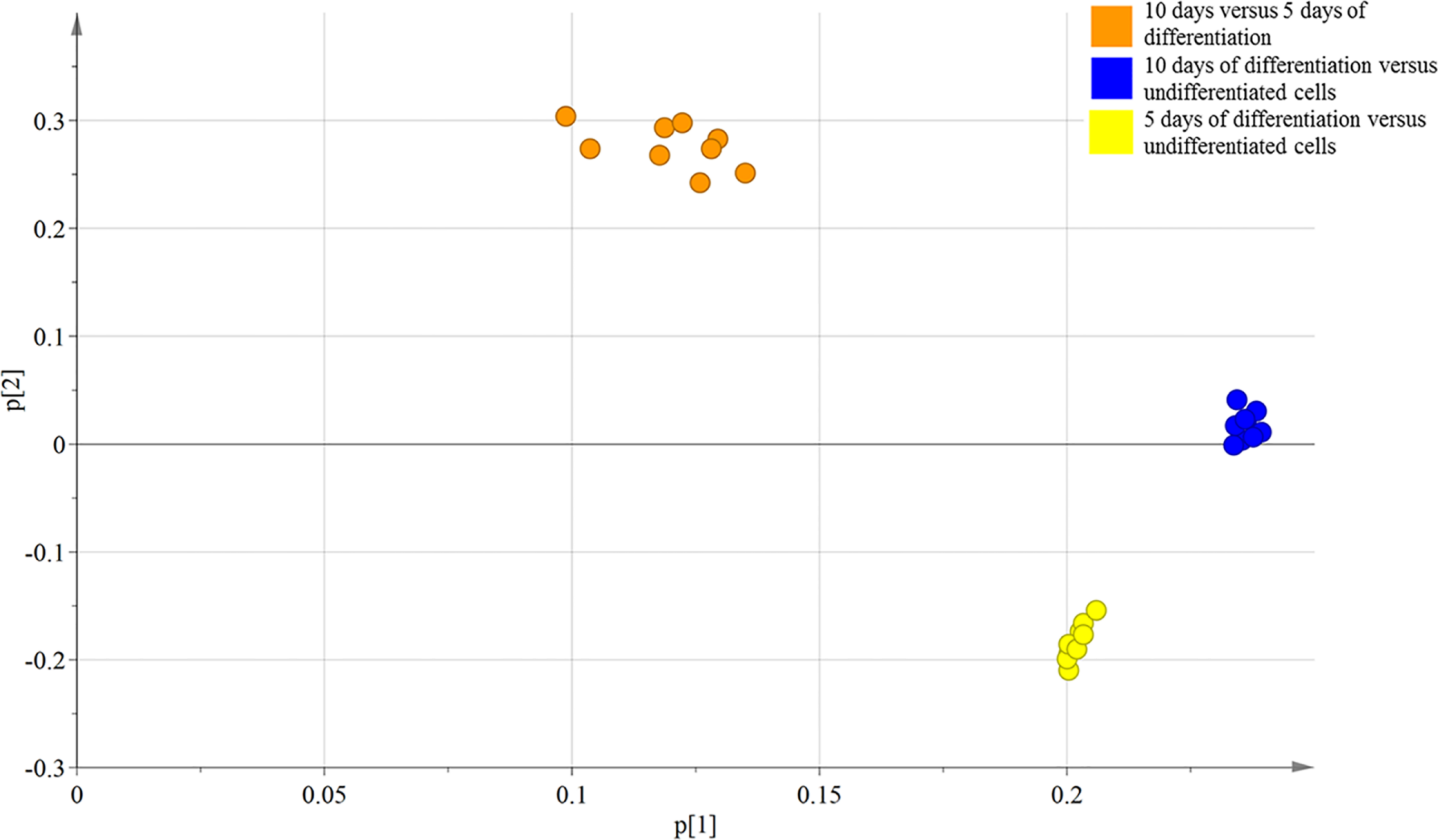
PCA plot of independent experimental seed-outs. The data clusters according to the different contrasts, i.e. 10 days vs 5 days of differentiation, 10 days vs undifferentiated, 5 days vs undifferentiated, showing robustness of the cell model as well as technical reproducibility. The first two principal components explained 72.5% of the information (variation) of the dataset (for PC1: 55.7%, for PC2: 16.8%).

C17.2 neural progenitor cells were used as a model for the developing nervous system. In this study, the cells were differentiated in serum-free N2 medium with NGF and BDNF for 5 or 10 days (S1A–S1C Fig). The C17.2 cells showed upregulated protein levels of both βIII-tubulin (a neuronal marker) and glial fibrillary acidic protein (GFAP, an astrocytic marker) as well as a downregulation of nestin (a marker for neural progenitor cells) during differentiation (S1 Fig). The ratio of neurite-bearing cells was approximately 20% for cells differentiated for 5 days and 35% for cells differentiated for 10 days, determined by counting cells on the phase contrast images (S1E Fig). Further characterization of the different neuronal phenotypes in the differentiated C17.2 cultures showed an upregulation of both Glutamate decarboxylase 1 (GAD1, a marker for gamma-amino butyric acid (GABA) neurons) and vesicular glutamate transporter 1 (vGluT1, a marker for glutamatergic neurons) (S1D Fig), whereas biomarkers for other neuronal phenotypes where not expressed (i.e. ChAT, TH and TPH2) (data not shown). Results from the microarray also showed that markers for neural stem cells were downregulated during differentiation (e.g. SOX1/3, NES and MKI67), however the results were not validated with RT-qPCR (Gene Expression Omnibus GSE97337). One approach for analysis of microarray data is by using PCA. The PCA analysis will test if the data are robust, i.e. if the data clusters or not. PCA is mathematically defined as an orthogonal linear transformation that transforms the multivariable data to a new coordinate system. In the new coordinate system, the largest variance by the projection of the data is presented in the first coordinate, called the first principal component (PC). The second largest variance is subsequently projected onto the second coordinate and so on. This reduces the dimensionality of the data while retaining most of the variation in the data set. Hence, samples can be plotted to visually assess similarities and differences between samples and determine whether or not samples can be grouped. The PCA plot of all the independent experiments (Fig 1) illustrates that the data clustered according to the different contrasts *i*.*e*. 10 days vs 5 days of differentiation, 10 days vs undifferentiated, 5 days vs undifferentiated, showing robustness of the cell model as well as technical reproducibility. The first two principal components explained 72.5% of the information (variation) of the dataset. The variance for PC1 was 55.7% and 16.8% for PC2.

**Fig 2:**
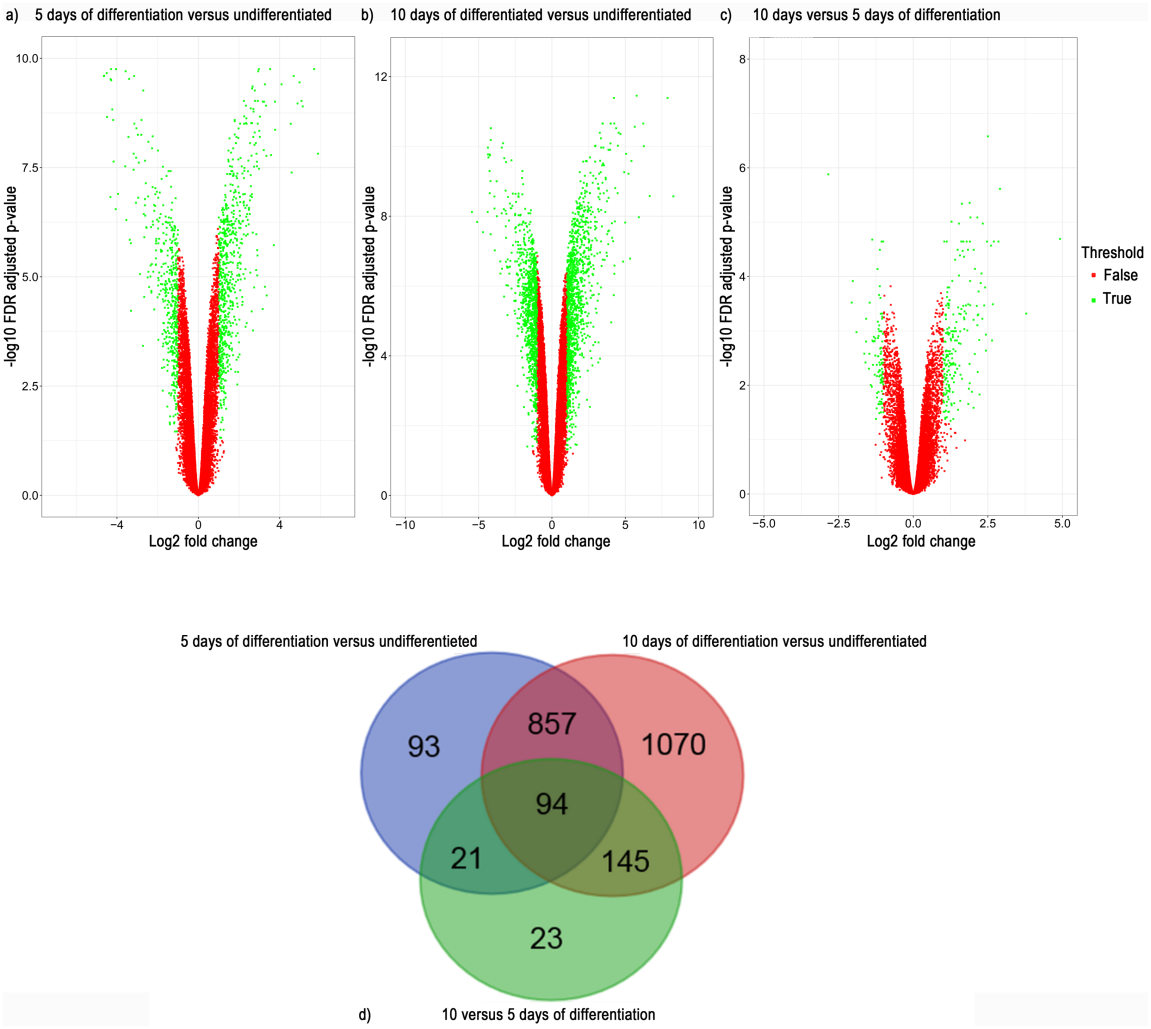
Volcano plot showing genes in and outside of cutoff values for differentially expressed genes (i.e. adjusted p-value ≤ 0.05 and absolute log2(fold change) >1). Red dots represent genes outside of the cutoff values and green dots represents differentially expressed genes at a) 5 days of differentiation vs undifferentiated cells b) 10 days of differentiation vs undifferentiated cells c) 10 days vs 5 days of differentiation d) Venn diagram showing overlap of differentially expressed genes between the different time points.

Volcano plots were used to visualize genome-wide gene expression. The differentiated cells (5 or 10 days of differentiation) were compared to undifferentiated cells (Fig 2a and 2b, respectively) or compared to each other (5 days vs 10 days of differentiation) (Fig 2c). Gene-wise fold change values (log2 scale) are plotted on the x-axis against FDR-adjusted significance values (negative log10 scale) on the y-axis. Genes that had an absolute log2(fold change) expression >1 together with a FDR-adjusted p-value ≤0.05 were defined as differentially expressed and selected for further analysis. The Venn diagram (Fig 2d) shows the number of DEGs that overlap between the different contrasts, *i*.*e*. undifferentiated cells, 5 days and 10 days of differentiation. The comparison between cells differentiated for 10 days and undifferentiated cells generated the largest number of DEGs, 2166 genes (1216 upregulated and 950 downregulated). The contrast between cells differentiated for 5 days and undifferentiated cells consisted of approximately half the number of DEGs, 1065 genes (665 upregulated and 400 downregulated). The contrast between cells differentiated for 10 days and cells differentiated for 5 days only resulted in 283 DEGs (192 upregulated and 91 downregulated), indicating that most changes occur during the first 5 days of differentiation. 94 genes overlapped between the 3 contrasts.

### Pathway analysis gives insight into the biological changes during neural differentiation

**Fig 3:**
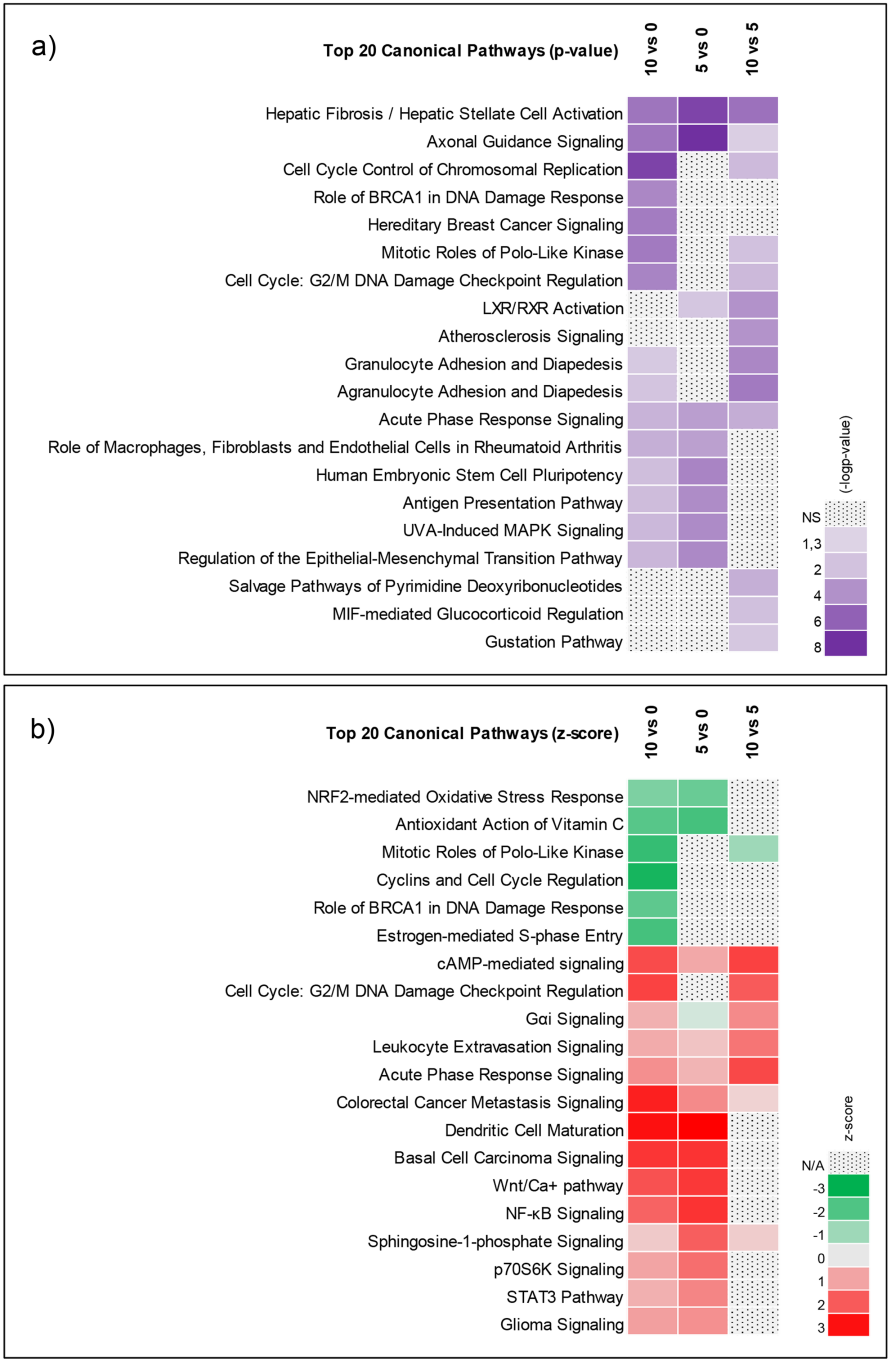
Canonical pathway analysis of differentially expressed genes using IPA. a) Top 20 Canonical pathways as per p-value b) Top 20 as per z-score (a measure of the predicted direction of the pathway activity).

Ingenuity Pathway Analysis (IPA) software was used to perform pathway analysis for the 3 contrasts (5 days of differentiation vs undifferentiated, 10 days of differentiation vs undifferentiated and 10 days of differentiation vs 5 days of differentiation). The top 20 pathways according to the level of significance and level of activation from each contrast are included in Fig 3. The top enriched pathway was (Hepatic) fibrosis, which was defined by genes related to the extracellular matrix and not by liver specific genes (as the name misleadingly suggests), suggesting matrix remodeling during neural differentiation [42]. Axonal guidance signaling pathway was highly significant, as indicated by enrichment in all 3 contrasts (Fig 3a and 3b). Heatmaps of the genes defining the axonal guidance signaling pathway are included (S2 Fig). Six of the genes curated in the axonal guidance signaling pathway were identified as important biomarkers for neural differentiation of the C17.2 cell line (BMP4, PLXNB3, PLXNA3, SLIT2, ROBO1 and NTN1) and hence, were included among the 30 biomarkers selected for validation. As indicated in Fig 3, the NRF2-oxidative stress response pathway together with the cyclin and cell cycle regulation pathway were predicted to be inhibited whereas G2/M DNA damage checkpoint regulation, acute phase response signaling and NF-kB pathways were predicted to be activated during neural differentiation.

### Identification and selection of potential biomarker genes for neural differentiation

**Fig 4:**
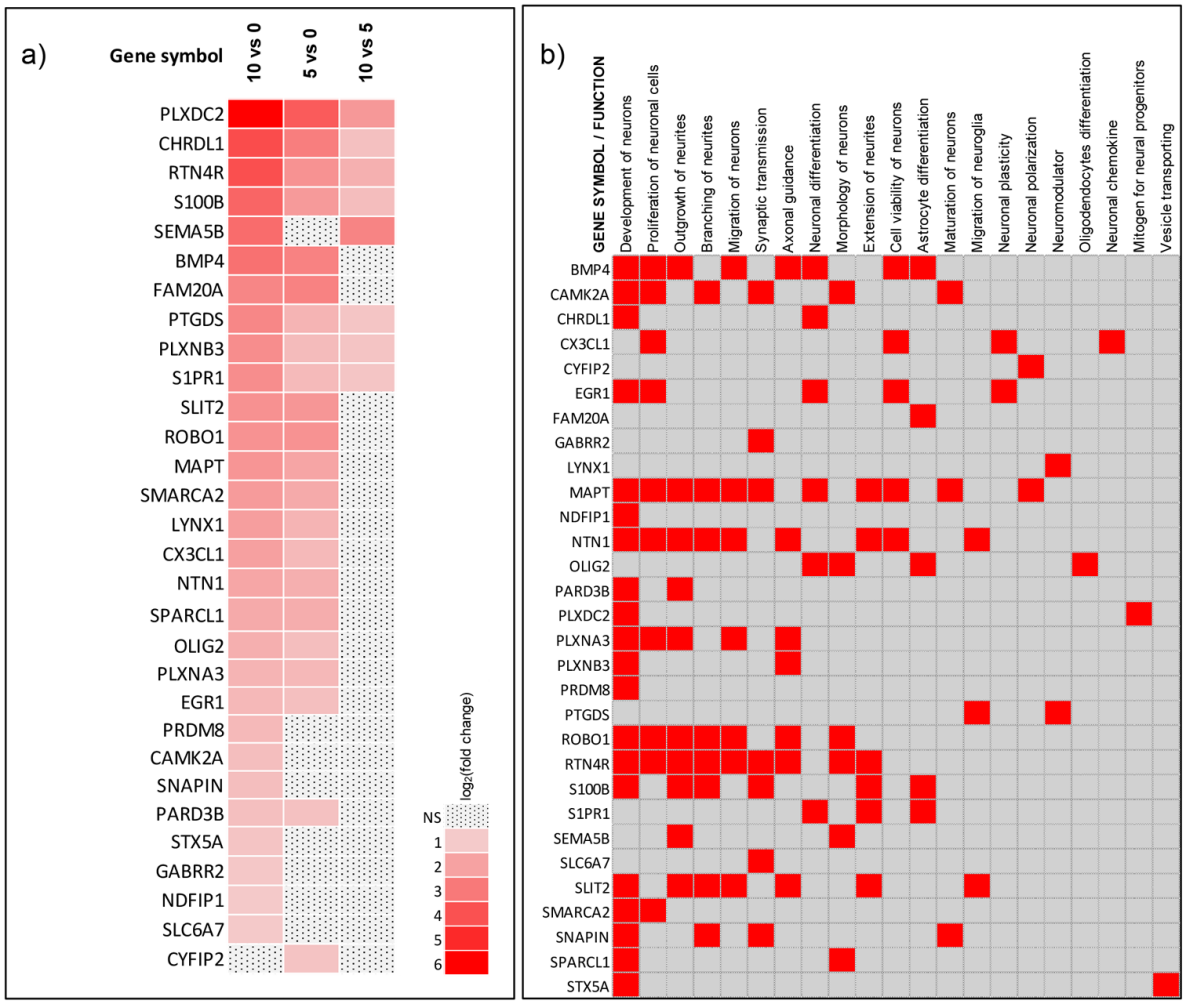
Mapping of the 30 genes selected as important for neural differentiation of the C17.2 cell line. a) Heatmap of the 30 selected genes for the contrasts 10 days of differentiation (Day 10) vs undifferentiated cells (Day 0), 5 days of differentiation (Day 5) vs undifferentiated and 10 days of differentiation vs 5 days of differentiation are illustrated. Genes are ordered according to average log2(fold change) in the contrast Day 10 vs Day 0. b) Map displaying the biological pathways/networks that the selected genes are involved in according to the IPA database as well as after manual review of published literature.

To select relevant biomarkers for neural differentiation of the C17.2 cell line, gene set enrichment analysis (GSEA) was performed on the genes selected as differentially expressed (http://software.broadinstitute.org/gsea/index.jsp). Neural specific enrichments lists (S1 Table) were carefully chosen, from which the 30 genes with the highest log2(fold change) values were selected to be further validated by using RT-qPCR analysis. Fig 4a illustrates the fold change values of the selected biomarkers, at different differentiation time points. The 30 selected biomarkers are involved in different neural processes, including neural development, axonal guidance, synaptic transmission as well as astrocyte and oligodendrocyte differentiation (S2 Table). In addition, we used this gene list and performed disease and function analyses with IPA in order to identify additional functions that these genes are correlated with. We then curated the functions from IPA and the functions from GSEA to generate a comprehensive heat map of the biological processes the selected genes are involved in (Fig 4b).

### The selected biomarker genes were successfully validated by RT-qPCR

**Fig 5:**
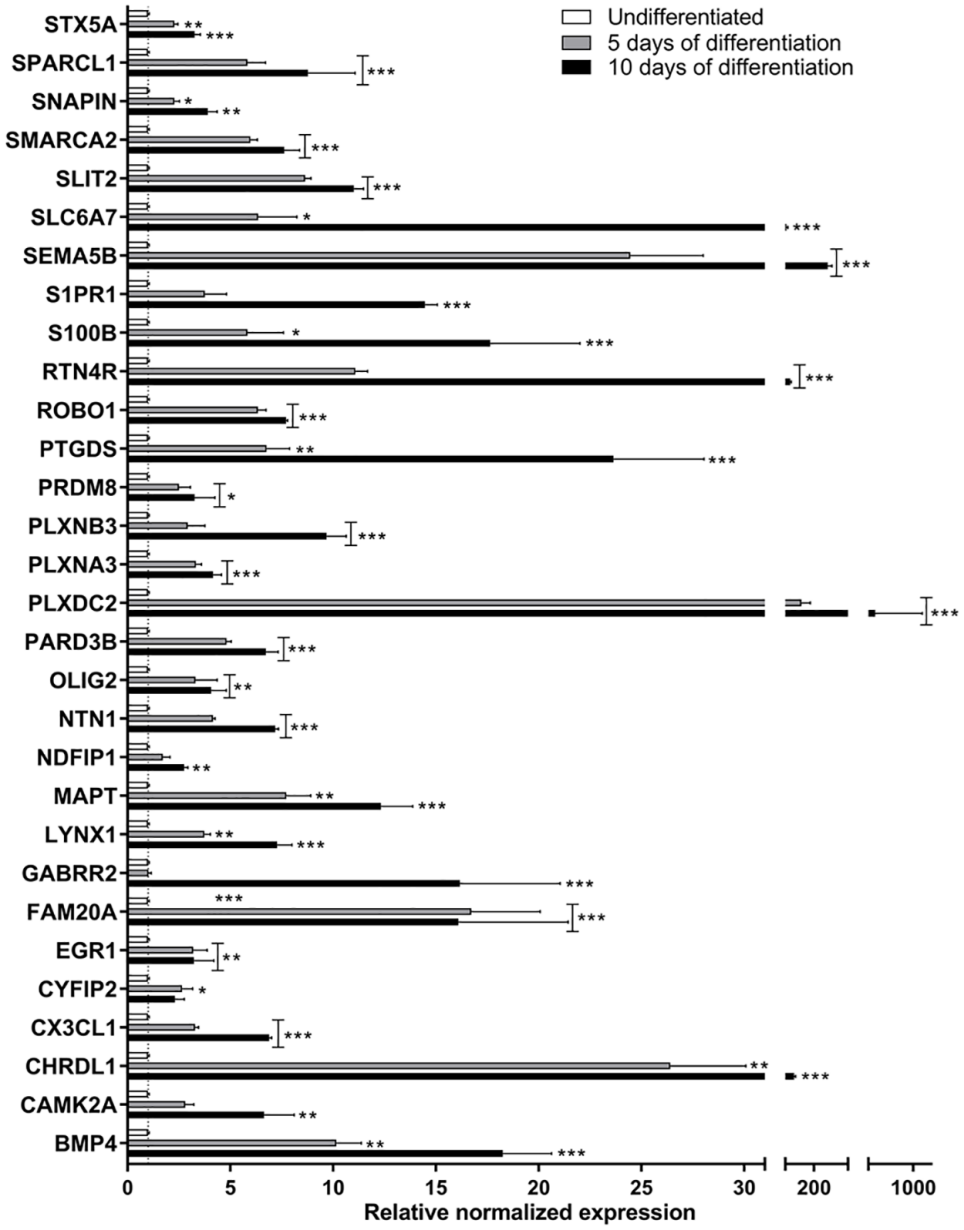
RT-qPCR validation of the 30 selected genes important for differentiation of the C17.2 cell line. The data are presented as the mean of 3 independent experiments. Results were analyzed using two-way ANOVA followed by Dunnett’s multiple comparisons test. The bars represent the mean ± SEM. *p ≤ 0.05, **p ≤ 0.01, ***p ≤ 0.001 compared to undifferentiated cells (unfilled bar).

The use of DNA microarrays has become a popular and helpful tool to perform discovery-based genomic research. However, studies comparing different microarray platforms have sometimes yielded conflicting results [43]. RT-qPCR is often denoted as the "golden standard" for gene expression measurements, generally due to its large dynamic range, its advantages in detection sensitivity, sequence specificity, high precision and reproducibility compared to other techniques. Hence, RT-qPCR has become the preferred method for quantifying gene expression as well as for independent validation of microarray results [44]. Hence, the 30 selected biomarkers were further validated with RT-qPCR (Fig 5). All 30 genes were significantly upregulated during differentiation as compared to undifferentiated cells, which was consistent with the microarray data.

Alterations in expression levels were normalized against the 3 reference genes; TBP, Hsp90ab and Rplp1, which fulfilled the set criteria (mentioned in the materials and methods section). They were further statistically validated using RT-qPCR with the help of the”target stability” function in the Bio-Rad CFX manager 3.1 software system. This function uses an iterative test of pairwise validation described by [45] (S3 Table).

### The expression of the selected biomarkers was altered by well-established neurotoxic compounds

**Fig 6:**
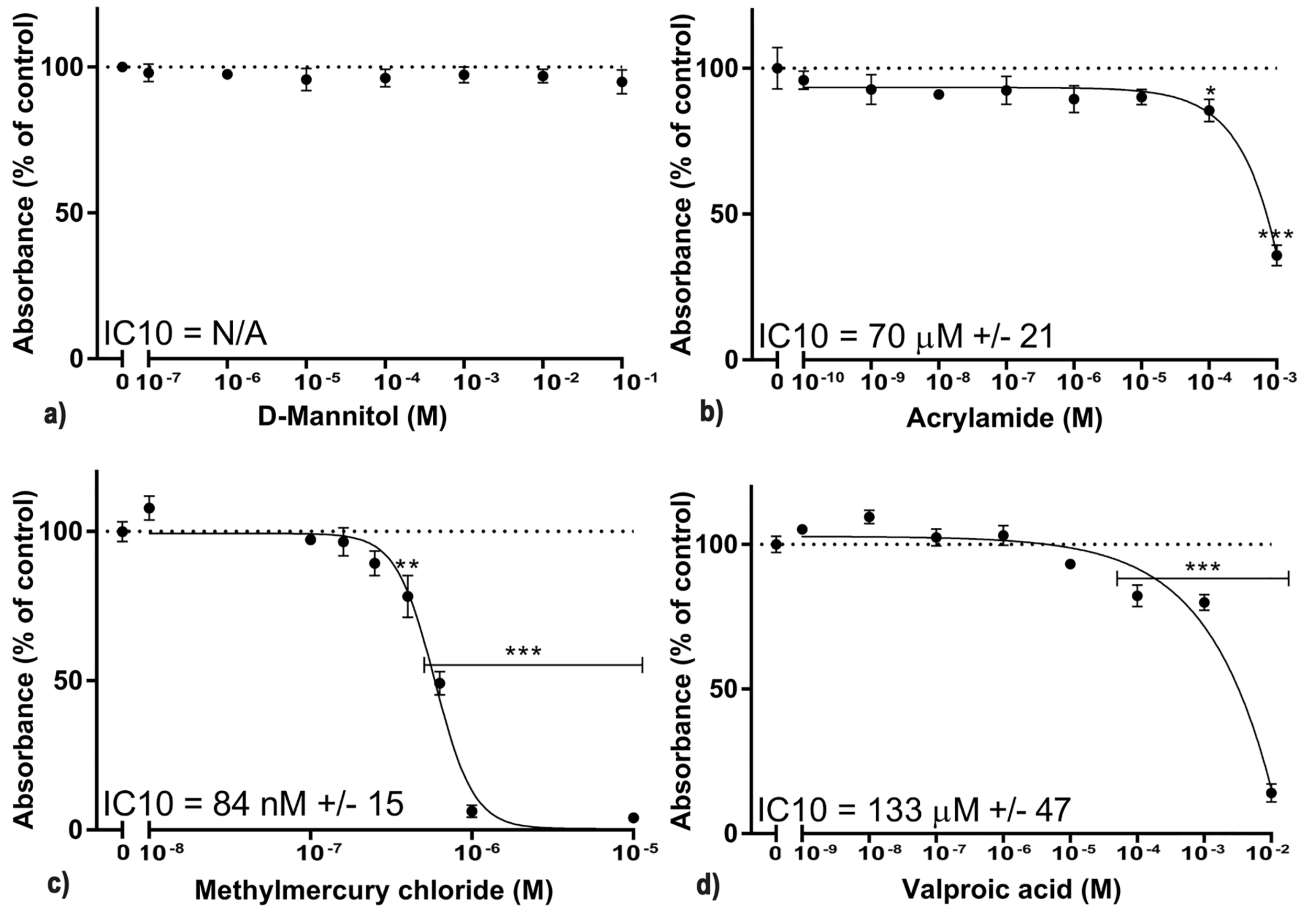
Cell viability of the C17.2 cells during exposure of a wide range of concentrations for four different compounds. The IC10 concentration was calculated and was further used to validate proof of concept of the 30 selected genes. Cells exposed to a) D-mannitol (negative control) b) acrylamide c) methylmercury chloride d) valproic acid sodium salt. The data are presented as the mean of 3 independent experiments preformed in hexaplicates. Results were analyzed using two-way ANOVA followed by Dunnett’s multiple comparisons test. The bars represent the mean ± SEM. *p ≤ 0.05, **p ≤ 0.01, ***p ≤ 0.001 compared to control (cells exposed to only cell medium). The inhibitory concentration 10% (IC10) was determined from nonlinear regression to fit the data to the log(inhibitor) vs response(variable slope) curve using the Hill slope (slope factor), equation Y = Bottom + (Top-Bottom)/(1+10^((LogIC10-X)*HillSlope)) (GraphPad Prism 7.02).

**Fig 7:**
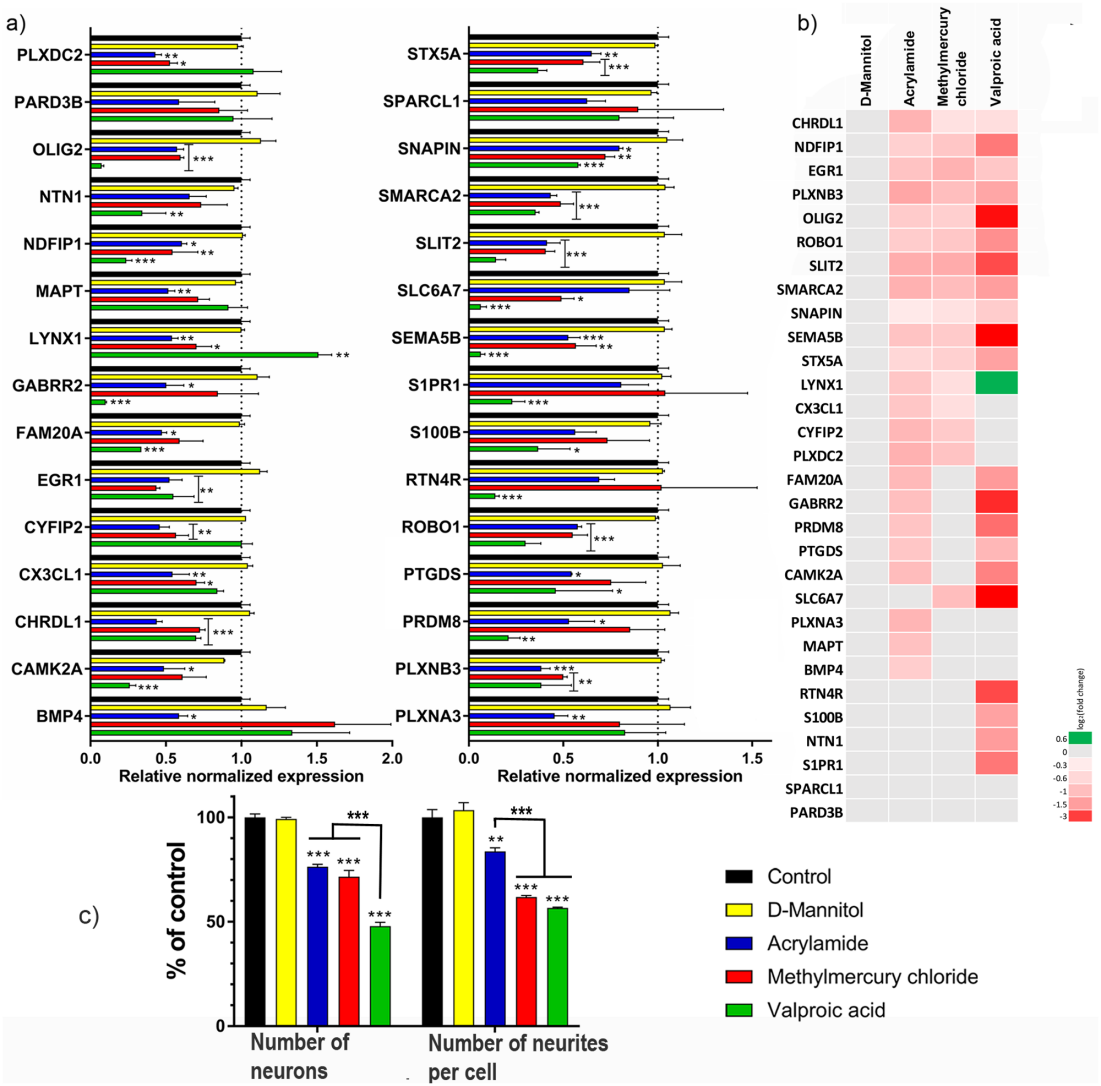
The effect of D-mannitol (negative control), acrylamide (ACR), methylmercury chloride (MeHg) and valproic acid sodium salt (VPA) on gene expression, the number of neurons and neurites per cell in differentiating C17.2 cells. a) RT-qPCR of all 30 genes after 10 days of differentiation and exposure to the IC10 of said compounds (70 μM of ACR, 90 nM of MeHg and 100 μM of VPA. D-mannitol did not show any cytotoxicity for the concentrations used, and 1 mM was chosen for cellular exposure) b) Heatmap of the 30 genes expression during exposure to the 4 compounds. The log2(fold change) for the contrasts as compared to the control (unexposed) are illustrated c) the number of neurons and the number of neurites per cell decreased after exposure to all 3 neurotoxic compounds. The data are presented as the mean of 3 independent experiments performed in duplicates. Results were analyzed using two-way ANOVA followed by Dunnett’s multiple comparisons test. The bars represent the mean ± SEM. *p ≤ 0.05, **p ≤ 0.01, ***p ≤ 0.001 compared to control (cells exposed to only cell medium) or between the 3 different compounds (ACR, MeHg and VPA).

To further validate the selected biomarkers for neural differentiation of C17.2 cells, we evaluated their expression following treatment with four different xenobiotics. As a common rule, organ type test systems should assess specific adverse events independent of general cytotoxicity [46]. For example, studying the inhibition of neurite outgrowth should not be performed at cytotoxic concentrations [15]. Commonly, concentrations ≤IC10 are considered as non-cytotoxic and used to study specific adverse events [14,47]. The AlamarBlue cell viability assay was used to estimate the IC10 for the C17.2 cells during 10 days of differentiation and exposure (Fig 6). A wide range of concentrations was studied in terms of general test quality control. The D-mannitol-exposed cell cultures displayed no cytotoxicity. Concentrations close to the mathematically calculated IC10 were selected. After exposure for 10 days during differentiation, the C17.2 cells were harvested and the selected biomarkers were analyzed with RT-qPCR. Phase contrast images captured immediately before harvesting showed that the morphology of the cells had changed, even at non-cytotoxic concentrations of the substances (S3 Fig). On a functional level, all 3 neurotoxic substances significantly reduced the number of neurons (Fig 7c). The number of neurites per neuron was also reduced for all 3 substances (Fig 7d). Out of the 3 substances, VPA reduced the number of neuronal cells and number of neurites per neuron the most. Twenty-eight of the 30 neuro-specific biomarkers were significantly altered by one or more of the 3 neurotoxic substances (Fig 7a and 7b).

## Discussion

Despite its high relevance, DNT is one of the least studied forms of toxicity [11]. Keeping up hazard assessment with the rapid production of new compounds is extremely time consuming and costly. Using *in vivo* models can be challenging due to many factors, *e*.*g*. species differences, extrapolation difficulties and complex mechanisms of toxicity. The use of *in vitro* and *in silico* models for toxicity screening is both cheaper, faster and more ethically attractive. Using a battery of different assays, looking at both general toxicity and more target-dependent toxicity, could also give a broader insight to the mechanism of toxicity for specific compounds. DNT can be particularly difficult to study due to the fact that the cellular effects do not generally result in cell death, but can be displayed as subtle changes in neuronal positioning, connectivity or morphology. In addition, the effects of DNT might not be measurable for a long time after the exposure of the chemical has ceased. For example, if the DNT-producing chemical is introducing epigenetic changes, the effects of the chemical can linger much longer than the actual exposure itself and could give rise to DNT over time [18]. One should also keep in mind that DNT is occurs in a tissue that is under constant remodeling, making the need for reliable controls extremely important [48].

The C17.2 progenitor cell line is a promising model to study DNT since it can be concurrently differentiated into a co-culture of both neurons and astrocytes [29]. In 2D cultures, the neurons differentiate and extend neurites on top of the proliferating neuroglial cells. The neuroglial cells provide neurotrophic support for the differentiating neurons and mimic the *in vivo* situation better than monocultures of differentiating neurons. Additionally, astrocytes have been shown to be able to modify the neural response to different toxic substances [49], therefore a co-culture of both neurons and astrocytes might give a more complete model of the toxicological response *in vivo*. The C17.2 cell line has also been used for DNT studies in the past to study DNT-introducing chemicals such as methylmercury, manganese and arsenic [50–52]. We have previously shown that the C17.2 cell model could be used to study ACR induced DNT by studying both mRNA and protein levels of BIII-tubulin and GFAP, viability, as well as the ratio of the different neural cell populations during differentiation [15].

The use of transcriptomics for toxicological studies has become a useful tool over the last decades [27]. Toxicogenomic approaches consist in evaluating gene expression changes in response to xenobiotic exposure and can be valuable for generating hypothesis regarding specific mechanisms of toxicity. For DNT, transcriptomics make it possible to identify chemicals that affect genes that are highly regulated during differentiation [20,53]. The correlation of gene expression patterns with toxicological endpoints is essential for prediction of toxicity by expression profiling. However, the use of whole genome array analysis to study DNT results in a huge amount of data and might be considered too expensive to ultimately be used for high throughput screening. Predefined gene sets, containing a minimal number of genes that are able to detect and classify DNT is preferable and has previously been shown to be able to identify DNT-inducing reference chemicals [23]. Hogberg et al. showed that altered mRNA levels for the neuronal markers NF-68 and NF-200 (covering the initial neurite outgrowth and the later stages of morphological maturation), N-methyl D-aspartate glutamate receptor and GABA receptor (main neuronal excitatory and inhibitory receptors) as wells as astrocytic markers GFAP and S100 calcium binding protein B (S100B), could be used for the initial identification of DNT effects and the underlying mechanisms of toxicity in rat cerebellar granule cells [23]. It is not possible to identify every DNT-inducing chemical by using a small set of predefined biomarkers. The use of *in silico* predictions of possible targets for new chemicals could help in the selection of relevant biomarkers to screen. Of course, one should keep in mind that choosing too few biomarkers could pose a risk to miss certain chemicals (identify false-negative chemicals).

The C17.2 cell model is suitable to use for high throughput screening since it generates robust and technical reproducible data. Robustness is a measure of a methods’ capacity to remain unaffected by small variations in method parameters and environmental conditions. Analyzing the robustness of a model provides an indication of its reliability during normal usage and should not be disregarded as it is of significant importance, especially for more complex systems such as DNT [46]. We have, with the help of whole genome array analysis, statistics and gene enrichment lists, selected and characterized 30 genes that are of high importance for differentiation of the C17.2 neural progenitor cells under the described conditions. The IPA library canonical pathways analysis identified the pathways which were the most significantly upregulated after 5 and 10 days of differentiation. Genes involved in various pathways were differentially expressed, even if some of them were not directly linked to neural differentiation. The top canonical pathway (hepatic fibrosis/hepatic stellate cell activation) consists of a multitude of genes involved in the remodeling of the extracellular matrix that occurs during differentiation [42] and can be seen in detail in supplementary data (S2 Fig). The alteration of the NRF2-mediated oxidative stress response indicates changes in reactive oxygen species (ROS) which govern the acquisition of the neural fate, from neural induction to the elaboration of axons during neural differentiation [54]. In addition, ROS can regulate redox sensitive transcription factors such as NFκB, AP-1 and can influence neurogenesis through modulation of the redox state of tyrosine phosphorylated proteins [55]. The Wnt/Ca^2+^ pathway was upregulated during differentiation and has previously been reported to play a role in the regulation of dendritic spines and synaptic strength through a mechanism involving signaling by Wnt-5a and Wnt -7a and further activation of CamKII [56].

The 30 selected genes are all involved in a wide range of diverse pathways. A wide spread of differential pathways increase the chances of different substances to be identified by the panel of biomarkers, since they will be able to identify substances with varied modes of action (MOA). We also tested if the biomarkers could detect DNT by exposing the cells to two known DNT-producing substances and one neurotoxic and possible DNT-producing substance. The two known positive controls have slightly different MOAs but were still identified by most of the biomarkers, showing the significance of these biomarkers for neural differentiation of the C17.2 cell line. ACR and MeHg, bind to thiol groups, predominantly the cysteine thiol group in the antioxidant glutathione, suggesting similar MOA for the two compounds [57]. Maintaining the right balance of reactive oxygen species has been shown to be crucial for regulation of self-renewal and differentiation in pluripotent cells [53]. It has been suggested that the ACR-induced neurotoxicity is mediated through axonopathy caused by initial distal nerve terminal damage and subsequent retrograde axon degeneration [33]. The DNT-inducing properties of ACR are still under investigation but studies have shown that prenatal and perinatal exposure to ACR decreases the average horizontal motor activity and auditory startle response in exposed rats [58]. We have previously shown that ACR downregulated the mRNA levels of markers for semi-mature neurons and astrocytes in differentiating C17.2 cells as well as attenuating differentiation of neurons *in vitro* in both SH-SY5Y and C17.2 cells at very low concentrations [15]. However, only few studies evaluated changes in mRNA expression during differentiation and ACR exposure. Nevertheless, in this study we observed that ACR significantly downregulated most of the selected biomarkers after exposure to a non-cytotoxic concentration. In coherence with our previous study, the results indicate that ACR might cause DNT.

Exposure to MeHg during development results in mental retardation including learning and behavioral deficits in both humans and mice [59]. Prenatal exposure has shown to cause disruption in the postnatal development of the glutathione antioxidant system [60]. There are also correlations between learning disabilities and increased DNA methylation and repressive histone modifications at the BDNF promoter in mice exposed to MeHg *in utero* [61]. *In vitro*, MeHg inhibited axonal outgrowth in PC12 cells through interfering with TrkA signaling after NGF stimulation [62,63]. Hence, it is not surprising that MeHg had an inhibitory effect on many of the selected biomarkers in a model where differentiation is partly driven by NGF. In coherence with earlier studies, the neurons in our model system were more sensitive to MeHg exposure than neuroglial cells and neural progenitor cells since the neurons accumulate more MeHg [64,65]. All 3 astrocytic biomarkers (FAM20A, S100B and S1PR1) were left unaffected by MeHg.

VPA exposure during pregnancy has been associated with a number of developmental abnormalities including neural tube defects, spina bifida, autism and bipolar disorder [66]. *In vitro* transcriptomics studies have shown that VPA downregulated several genes involved in neural differentiation at the same time as upregulating genes involved in neural precursor proliferation [23]. These attributes are thought to be due to the fact that VPA inhibits histone deacetylase enzyme activity and may therefore disturb normal gene transcription [67]. In coherence with earlier studies, the C17.2 cells were also affected by VPA (Fig 7a). Most genes were downregulated but there was one gene that was upregulated (LYNX1), displaying the wide array of disturbance in gene expression following VPA exposure.

Additionally to downregulating most of the selected biomarkers for neural differentiation, all 3 of the substances significantly reduced the ratio of neurons in the cultures as well as the number of neurites per cell. The fact that the downregulation of the biomarkers also results in structural consequences further strengthens the importance of these biomarkers for neural differentiation of the C17.2 cells and that disruption of the expression levels results in functional implications for the cells during differentiation. VPA was the substance with the greatest effect on the ratio of the neural populations as well as the number of neurites per cell followed by MeHg and ACR. There was a statistically significant difference between the numbers of neurons in cultures exposed to ACR or MeHg compared to cultures exposed to VPA. The number of neurites per cell was significantly different between cultures exposed to ACR in comparison to cultures exposed to MeHg or VPA. It seems like MeHg did not affect the number of neurons as much as it affected the number of neurites per cell. VPA was also the substance that affected the selected biomarkers the most in terms of log2(fold change) values, showing that there is a correlation between the biomarkers and the structural readouts. In parallel with the differentiating cell cultures prepared for the microarray analysis, differentiating cells were exposed to 1 μM of ACR and analyzed in the whole genome microarray. Cells exposed to 1 μM of ACR during differentiation for 5 or 10 days showed no change in gene expression for the selected biomarkers (data not shown). At this concentration of ACR, there was no significant reduction in the number of neurites per cell. This indicates that there is a correlation between a significant downregulation of the mRNA biomarkers and functionality of the differentiating cells, which further validates the model. None of the 3 substances changed the mRNA levels of three generally expressed genes (HSP90ab1, Rplp1 and TBP) during the differentiation, which indicates that the downregulation seen in the neural biomarkers are not due to a general downregulation of all genes in the cells.

Out of the 30 selected biomarkers, there were 11 biomarkers that were downregulated by all 3 substances. These 11 biomarkers are involved in wide spread of categories for neural differentiation. As a preliminary prediction model it would be interesting to see if a significant downregulation of these 11 biomarkers would be a good alert for DNT. However, to be able to draw any such conclusions, a large set of test compounds need to be tested to validate the system.

There were two biomarkers that were not affected by any of the 3 substances, PARD3B and SPARCL1. During the first few weeks of postnatal development, SPARCL1 (SPARC-Like protein 1/Hevin) is highly expressed in astrocytes [68], but is also present to some extent in neurons [69]. Secretion of Hevin from astrocytes has been shown to play an important role during synapse development [70]. One reason why the levels of SPARCL1 was unaffected might be that Hevin is generated and secreted mostly by astrocytes. In general, astrocytes have a higher ability to metabolize xenobiotics, for example upregulating anti-oxidant systems that can protect against ACR and MeHg [71], leaving them less affected by the toxic insult than the neurons. PARD3B (Par-3 Family Cell Polarity Regulator Beta/PARD3) is a member of the PARD adaptor proteins and has shown to be involved in axon-dendrite polarization as well as neuronal migration [72]. However, there is no genetic loss-of function evidence establishing that PARD3B is required for axon specification [73]. There is so far no evidence that any of the selected xenobiotics are interfering directly with PARD3, which might explain why it is not affected.

The basic helix-loop-helix transcription factor OLIG2 was also downregulated by all 3 substances. The expression of OLIG2 does not necessarily indicate the presence of oligodendrocytes in the cultures. OLIG2 has been shown to be a multifunctional regulator of self-renewal in neural stem cells and seems to have a role in maintaining proliferation as well as repress quiescence genes [74].

The fact that the selected biomarkers are part of a wide array of different pathways, further strengthens the possibility to detect toxic insult from xenobiotics with unknown MOAs. Moving forward, it would be possible to further develop the model to investigate the exact mechanism of action of a chemical of interest. For example, platforms of biomarkers could be selected that are involved in specific functions in the cell that are predicted to be affected by the chemical of interest. This possibility has been utilized in the Genomic Allergen Rapid Detection (GARD) assay, which presently undergoes validation for acceptance in the OECD test guideline as an indicator of skin sensitization [75]. It is also possible to study the gene expression during development with this model by performing washout experiments, or shorter exposure times to see during which time periods the cells are most susceptive to a chemical.

The relevance of toxicogenomic approaches in safety testing is widely recognized. According to a revision of non-clinical safety studies, it was concluded that significantly regulated transcripts can serve as robust biomarkers of toxicity [76]. The study showed that there was poor correlation with histopathological findings, however, transcriptomics showed to be a very sensitive marker and often preceded more traditional endpoints.

In conclusion, from a whole genome set of mRNA transcripts we identified 30 biomarkers, which were significantly affected during neural differentiation of the C17.2 neural progenitor cell line. The biomarkers correlated to genes that are involved in neural networks according to the IPA database as well as manual review of published literature. They were selected for further validation due to their strong upregulation during neural differentiation and without further bias. The biomarkers cover most of the important categories of neural differentiation such as neurogenesis, axonogenesis, axonal guidance, astrocyte- and oligodendrocyte differentiation and neuronal connectivity, further increasing the chance of identifying substances with a wide range of MOAs. Using a set of mRNA biomarkers, instead of the whole genome, makes this model affordable and applicable for high-throughput screening. The method can be screened with optimized primers using RT-qPCR, a method that most laboratories have access to. The C17.2 cell line can be differentiated in a 2D-system without additional plate coating and with the addition of only two neurotrophic factors, making it a simple, fast and cheap model to use. The use of such models could help speed up the initial screening of substances, possibly indicating alerts that needs to be further studied in more sophisticated models.

## Supporting information

S1 Fig Neural progenitor cells C17.2 during differentiation. A) Undifferentiated neural progenitor cells after 3 days in culture B) 5 days of differentiation C) 10 days of differentiation. The scale bars represent 50 μm in all images. D) mRNA expression of GAD1 and vGluT1 during differentiation of the C17.2 cells, illustrating presence of GABAergic and glutamatergic neurons in the culture. In short, the cells were harvested and centrifuged at 500g for 5 min and stored in -80°C until mRNA extraction. The extraction was performed using GeneJET^®^ RNA Purification kit according to manufacturer’s instructions. Concentration of total RNA was determined by a NanoPhotometer^™^ P-class (IMPLEN GmbH) followed by reverse transcription of total RNA using RevertAid^®^ Minus First Strand cDNA Synthesis Kit. For quantitative real-time RT-PCR, 140 ng cDNA was used as a template together with Maxima^®^ SYBR Green/Fluorescein qPCR Master Mix (2x). Gene expression levels were measured by using the MyiQ^®^2 Two-color Real-Time PCR Detection System (Bio-Rad laboratories) and genes were normalized against TATA box binding protein (TBP). All kits and DNase1 were purchased from Fermentas, (Fischer Scientific) and performed according to instructions from the manufacturer. Primer sequences used were as follows: GAD1 forward; ACAAACTCTCAGCGGCATAGAAAGGG, reverse; AGCACGCCCATCATCTTGTGAG, vGluT1 forward; GTCCATGGTCAACAACAGCACAAC reverse; AGTTGAACTGGGCTTTCTGCAC, TBP forward; GAATTGTACCGCAGCTTCAAAA reverse; AGTGCAATGGTCTTTAGGTCAAGTT. E) Number of neurite-bearing cells compared to the total number of cells in the cultures after 5 and 10 days of differentiation F) Western blot of nestin (a marker for neural progenitor cells), βIII-tubulin (a neuronal marker) and glial fibrillary acidic protein (GFAP, an astrocytic marker). The cells were lysed in a hypotonic buffer containing NP-40. Twenty μg of total protein (determined with the DC Protein Assay, BioRad) were separated in 10% SDS- poly-acrylamide gels. The proteins were subsequently transferred to nitrocellulose membranes and hybridized with primary antibodies diluted accordingly: βIII-tubulin (ab18207) 1:5000, nestin (ab6142) 1:200 and GFAP (ab7260) 1:1000 (all from Abcam) and β-actin (sc-1616) 1:5000 (Santa Cruz). Horse radish peroxidase-conjugated anti-rabbit IgG (NA934 V) 1:3000 and anti-mouse IgG (NA931 V) 1:3000 (Amersham) and anti-goat IgG (sc-2020) 1:3000 (Santa Cruz) were used as secondary antibodies. Densitometric analysis of visual blots was performed using Image Gauge 3.46 program (Fujifilm Co. Ltd.). Results were analyzed using one-way ANOVA followed by Dunnett’s multiple comparisons test. The bars represent the mean ± SEM. *p ≤ 0.05, **p ≤ 0.01, ***p ≤ 0.001 for each biomarker compared to undifferentiated cells (unfilled/white bar).
(TIF)

S2 Fig Heatmap of the genes included in the axonal guidance signaling pathway. The log2(fold change) for the contrasts Day 10 (10 days of differentiation) vs Day 0 (undifferentiated cells cultured for 3 days), Day 5 (5 days of differentiation) vs Day 0 and Day 10 vs Day 5 are illustrated. Genes are ordered according to average log2(fold change) in the contrast Day 10 vs Day 0.
(TIF)

S3 Fig Phase contrast images taken same day as harvesting after 10 days of differentiation and exposure to the IC10 of the 4 different substances. A) Control B) D-Mannitol 1 mM C) Acrylamide 70 μM D) Methylmercury chloride 0.09 μM E) Valproic acid sodium salt 100 μM. The scale bars represent 50 μm in all images. F) Number of neurites per cell after 10 days of differentiation with different concentrations of ACR. Results were analyzed using one-way ANOVA followed by Dunnett’s multiple comparisons test. The bars represent the mean ± SEM. *p ≤ 0.05 compared to undifferentiated cells (unfilled/white bar).
(TIF)

S4 Fig GO enrichment analysis of the 30 most prominent/significant genes for neural differentiation of the C17.2 cell line. (TIF)

S1 Table Gene lists used for gene enrichment analysis for selection of genes important for differentiation of the C17.2 cell line. (PDF)

S2 Table The 30 selected genes including their description, protein function, the gene set enrichment list they were curated from and references. (PDF)

S3 Table Target stability function analysis of the three reference genes using the Bio-Rad CFX manager 3.1 software system. This function uses an iterative test of pairwise validation described by Vandesompele et al., 2002 [45]. Recommended coefficient variance should be <0.25 and M value should be <0.5 for homogenous samples.
(PDF)
