# Supplementary figures and images for: Whole genome microarray analysis of neural progenitor C17.2 cells during differentiation and validation of 30 neural mRNA biomarkers for estimation of developmental neurotoxicity
Source: PLoS One. 2017 Dec 20;12(12):e0190066. doi: 10.1371/journal.pone.0190066 (PMC5738075; doi:10.1371/journal.pone.0190066)

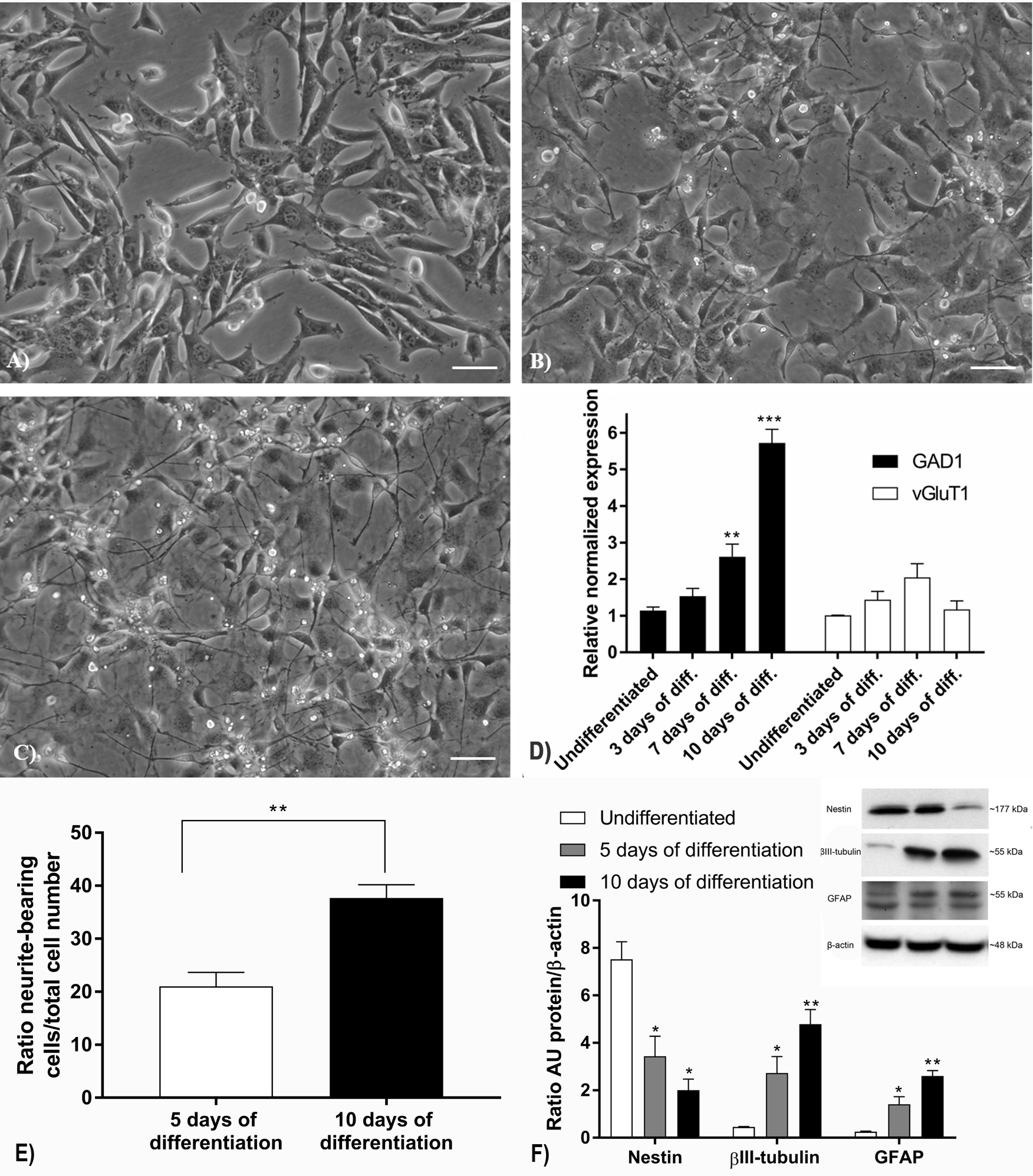

Supplement: S1 Fig — A) Undifferentiated neural progenitor cells after 3 days in culture B) 5 days of differentiation C) 10 days of differentiation. The scale bars represent 50 μm in all images. D) mRNA expression of GAD1 and vGluT1 during differentiation of the C17.2 cells, illustrating presence of GABAergic and glutamatergic neurons in the culture. In short, the cells were harvested and centrifuged at 500g for 5 min and stored in -80°C until mRNA extraction. The extraction was performed using GeneJET® RNA Purification kit according to manufacturer’s instructions. Concentration of total RNA was determined by a NanoPhotometer™ P-class (IMPLEN GmbH) followed by reverse transcription of total RNA using RevertAid® Minus First Strand cDNA Synthesis Kit. For quantitative real-time RT-PCR, 140 ng cDNA was used as a template together with Maxima® SYBR Green/Fluorescein qPCR Master Mix (2x). Gene expression levels were measured by using the MyiQ®2 Two-color Real-Time PCR Detection System (Bio-Rad laboratories) and genes were normalized against TATA box binding protein (TBP). All kits and DNase1 were purchased from Fermentas, (Fischer Scientific) and performed according to instructions from the manufacturer. Primer sequences used were as follows: GAD1 forward; ACAAACTCTCAGCGGCATAGAAAGGG, reverse; AGCACGCCCATCATCTTGTGAG, vGluT1 forward; GTCCATGGTCAACAACAGCACAAC reverse; AGTTGAACTGGGCTTTCTGCAC, TBP forward; GAATTGTACCGCAGCTTCAAAA reverse; AGTGCAATGGTCTTTAGGTCAAGTT. E) Number of neurite-bearing cells compared to the total number of cells in the cultures after 5 and 10 days of differentiation F) Western blot of nestin (a marker for neural progenitor cells), βIII-tubulin (a neuronal marker) and glial fibrillary acidic protein (GFAP, an astrocytic marker). The cells were lysed in a hypotonic buffer containing NP-40. Twenty μg of total protein (determined with the DC Protein Assay, BioRad) were separated in 10% SDS- poly-acrylamide gels. The proteins were subsequently transferred to nitrocellulose memb [file pone.0190066.s001.tif]

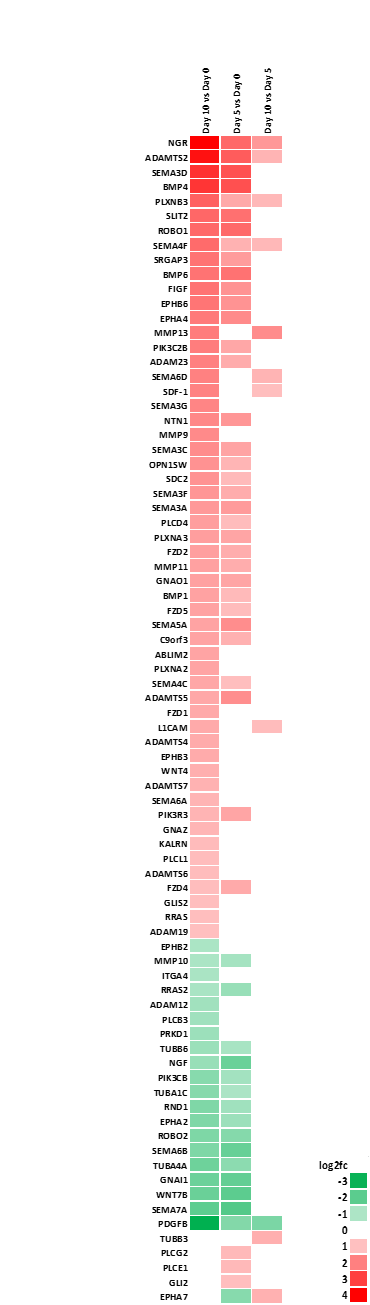

Supplement: S2 Fig — The log2(fold change) for the contrasts Day 10 (10 days of differentiation) vs Day 0 (undifferentiated cells cultured for 3 days), Day 5 (5 days of differentiation) vs Day 0 and Day 10 vs Day 5 are illustrated. Genes are ordered according to average log2(fold change) in the contrast Day 10 vs Day 0. (TIF) [file pone.0190066.s002.tif]

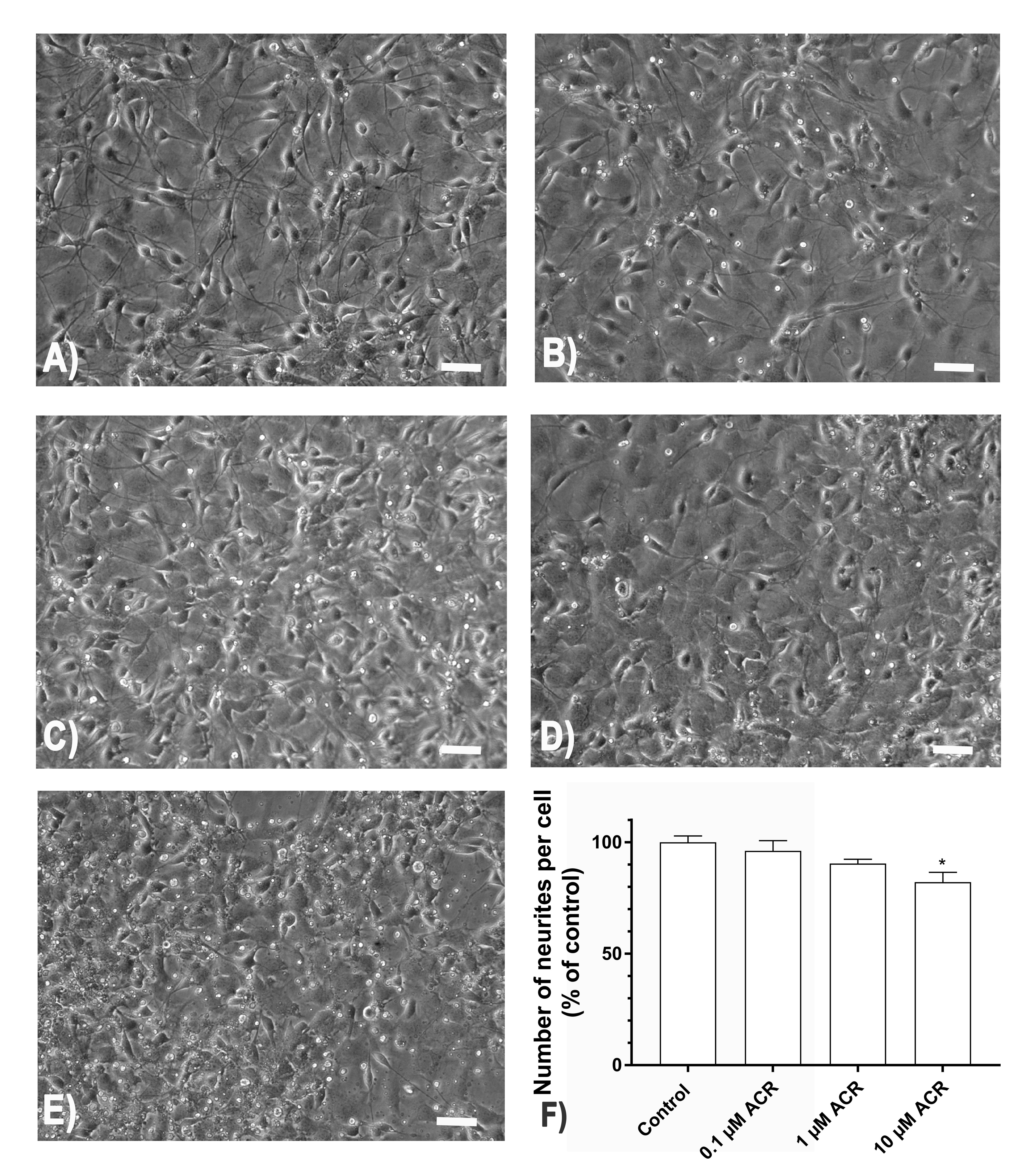

Supplement: S3 Fig — A) Control B) D-Mannitol 1 mM C) Acrylamide 70 μM D) Methylmercury chloride 0.09 μM E) Valproic acid sodium salt 100 μM. The scale bars represent 50 μm in all images. F) Number of neurites per cell after 10 days of differentiation with different concentrations of ACR. Results were analyzed using one-way ANOVA followed by Dunnett’s multiple comparisons test. The bars represent the mean ± SEM. *p ≤ 0.05 compared to undifferentiated cells (unfilled/white bar). (TIF) [file pone.0190066.s003.tif]
