# Supplementary material for: Whole genome microarray analysis of neural progenitor C17.2 cells during differentiation and validation of 30 neural mRNA biomarkers for estimation of developmental neurotoxicity
Source: PLoS One. 2017 Dec 20;12(12):e0190066. doi: 10.1371/journal.pone.0190066 (PMC5738075; doi:10.1371/journal.pone.0190066)
Supplement: S1 Table — (PDF) [file pone.0190066.s005.pdf]

S1 Table. Gene lists used for gene enrichment analysis for selection of genes important for differentiation of the C17.2 cell line.

**Gene set enrichment analysis list using Gene set enrichment analysis http://software.broadinstitute.org/gsea/index.jsp**
1. LEIN_OLIGODENDROCYTE_MARKERS http://www.broadinstitute.org/gsea/msigdb/cards/LEIN_OLIGODENDROCYTE_MARKERS.html
2. GOBERT_OLIGODENDROCYTE_DIFFERENTIATION_UP http://www.broadinstitute.org/gsea/msigdb/cards/GOBERT_OLIGODENDROCYTE_DIFFERENTIATION_UP
3. GOBERT_OLIGODENDROCYTE_DIFFERENTIATION_DN http://www.broadinstitute.org/gsea/msigdb/cards/GOBERT_OLIGODENDROCYTE_DIFFERENTIATION_DN.html
4. LEIN_ASTROCYTE_MARKERS http://www.broadinstitute.org/gsea/msigdb/cards/LEIN_ASTROCYTE_MARKERS.html
5. CAHOY_ASTROCYTIC http://www.broadinstitute.org/gsea/msigdb/cards/CAHOY_ASTROCYTIC.html
6. LEIN_NEURON_MARKERS http://www.broadinstitute.org/gsea/msigdb/cards/LEIN_NEURON_MARKERS.html
7. CAHOY_NEURONAL http://www.broadinstitute.org/gsea/msigdb/cards/CAHOY_NEURONAL.html
8. LEIN_LOCALIZED_TO_DISTAL_AND_PROXIMAL_DENDRITES http://www.broadinstitute.org/gsea/msigdb/cards/LEIN_LOCALIZED_TO_DISTAL_AND_PROXIMAL_DENDRITES.html
9. GO_SYNAPSE http://software.broadinstitute.org/gsea/msigdb/cards/GO_SYNAPSE.html
10. LEIN_LOCALIZED_TO_PROXIMAL_DENDRITES http://www.broadinstitute.org/gsea/msigdb/cards/LEIN_LOCALIZED_TO_PROXIMAL_DENDRITES.html
11. WONG_ADULT_TISSUE_STEM_MODULE http://software.broadinstitute.org/gsea/msigdb/cards/WONG_ADULT_TISSUE_STEM_MODULE.html
12. REACTOME_NEURONAL_SYSTEM https://bioinfo.uth.edu/SZGR1/showGenesByPathway.do?pathway=p874
13. LEIN_MIDBRAIN_MARKERS http://software.broadinstitute.org/gsea/msigdb/cards/LEIN_MIDBRAIN_MARKERS.html
14. NEUROGENESIS http://software.broadinstitute.org/gsea/msigdb/cards/NEUROGENESIS.html
15. AXON http://software.broadinstitute.org/gsea/msigdb/cards/AXON.html
16. CAHOY_OLIGODENDROCUTIC http://software.broadinstitute.org/gsea/msigdb/cards/CAHOY_OLIGODENDROCUTIC.html
17. LEIN_CEREBELLUM_MARKERS http://software.broadinstitute.org/gsea/msigdb/cards/LEIN_CEREBELLUM_MARKERS.html
18. ESC_V6.5_UP_EARLY.V1_UP http://software.broadinstitute.org/gsea/msigdb/cards/ESC_J1_UP_EARLY.V1_UP.html
19. MATZUK_EMBRYONIC_GERM_CELL http://software.broadinstitute.org/gsea/msigdb/cards/MATZUK_EMBRYONIC_GERM_CELL.html
20. AXONOGENESIS http://software.broadinstitute.org/gsea/msigdb/cards/AXONOGENESIS.html
