# Supplementary material for: Whole genome microarray analysis of neural progenitor C17.2 cells during differentiation and validation of 30 neural mRNA biomarkers for estimation of developmental neurotoxicity
Source: PLoS One. 2017 Dec 20;12(12):e0190066. doi: 10.1371/journal.pone.0190066 (PMC5738075; doi:10.1371/journal.pone.0190066)
Supplement: S2 Table — (PDF) [file pone.0190066.s006.pdf]

S2 Table. The 30 selected genes including their description, protein function, gene set enrichment list they were curated from and references.

Gene	Gene description	Protein function/Biological process	Gene set enrichment list
BMP4	Bone Morphogenetic Protein 4	Neurogenesis, Axonal guidance	MATZUK_EMBRYONIC_GERM_CELL
CAMK2A	Calcium/Calmodulin-Dependent Protein Kinase II Alpha	Synaptic transmission	LEIN_NEURON_MARKERS
CHRDL1	Chordin-Like Protein 1	Neurogenesis, Neuronal differentiation	CAHOY_ASTROCYTIC
CX3CL1	Fractalkine	Neuronal chemokine, Synaptic plasticity	LEIN_NEURON_MARKERS
CYFIP2	Cytoplasmic FMR1 Interacting Protein 2	Neuronal connectivity, Neuronal polarization	LEIN_NEURON_MARKERS, AXONOGENESIS
EGR1	Early Growth Response protein 1	Neuronal plasticity, Neuronal differentiation	LEIN_NEURON_MARKERS
FAM20A	Family With Sequence Similarity 20, Member A	Gene up-regulated in astrocytes	CAHOY_ASTROCYTIC
GABRR2	Gamma-Aminobutyric Acid A Receptor, Rho 2	Synaptic transmission	GO_SYNAPSE
LYNX1	Ly-6/Neurotoxin-like protein 1	Neuromodulator	LEIN_LOCALIZED_TO_PROXIMAL_DENDRITES
MAPT	Microtubule-Associated Protein Tau	Axonogenesis, Neuronal polarity	NEUROGENESIS, AXONOGENESIS
NDFIP1	Nedd4 Family-Interacting Protein 1	Neurogenesis, Development of dendrites and spines	LEIN_NEURON_MARKERS
NTN1	Netrin-1	Axonal guidance, Neuronal migration	LEIN_CEREBELLUM_MARKERS
OLIG2	Oligodendrocyte Transcription Factor 2	Oligodendrocyte differentiation, Neural stem cell proliferation	LEIN_OLIGODENDROCYTE_MARKERS
PARD3B	Par-3 Family Cell Polarity Regulator Beta	Axonogenesis, Neurogenesis, Neuronal migration	NEUROGENESIS, AXONOGENESIS
PLXDC2	Plexin Domain-Containing Protein 2	Mitogen for neural progenitors, Neurogenesis, Neuronal connectivity	GOBERT_OLIGODENDROCYTE_DIFFERENTIATION_DN
PLXNA3	Plexin-A3	Axonal guidance, Neuronal migration	GOBERT_OLIGODENDROCYTE_DIFFERENTIATION_DN
PLXNB3	Plexin B3	Neurogenesis, Axonal guidance	CAHOY_OLIGODENDROCUTIC
PRDM8	PR Domain Zink Finger Protein 8	Neuronal development	CAHOY_NEURONAL
PTGDS	Prostaglandin D2 Synthase 21kDa (Brain)	Neuromodulator and trophic factor in the CNS	LEIN_MIDBRAIN_MARKERS
ROBO1	Roundabout homolog 1	Axonal guidance, Neuronal migration	NEUROGENESIS, AXONOGENESIS

RTN4R	Reticulon-4 Receptor/Nogo-66 receptor	Axonogenesis	NEUROGENESIS
S100B	S100 Calcium Binding Protein B	Astrocyte differentiation, Axonogenesis	NEUROGENESIS, AXONOGENESIS
S1PR1	Sphingosine-1-Phosphate Receptor 1	Neurogenesis, Glial migration and Astrocyte proliferation	LEIN_ASTROCYTE_MARKERS
SEMA5B	Semaphorin-5B	Regulation of synapse density, Axonal guidance	GOBERT_OLIGODENDROCYTE_DIFFERENTIATION_DN
SLC6A7	Sodium-Dependent Proline Transporter	Synaptic transmission	CAHOY_NEURONAL
SLIT2	Slit Homolog 2 protein	Axonal guidance, Neuronal migration	NEUROGENESIS, AXONOGENESIS
SMARCA2	Transcription Activator SNF2L2	Neurogenesis, Neural stem cell proliferation	LEIN_NEURON_MARKERS
SNAPIN	SNAP-Associated Protein Snapin	Synaptic transmission, Synaptic vesicle recycling	GOBERT_OLIGODENDROCYTE_DIFFERENTIATION_DN
SPARCL1	SPARC-Like protein 1 (Hevin)	Secreted by astrocytes, Neuromodulator	LEIN_ASTROCYTE_MARKERS
STX5A	Syntaxin-5	Neuronal development, Vesicle transporting	REACTOME_NEURONAL_SYSTEM
