# Supplementary material for: Whole genome microarray analysis of neural progenitor C17.2 cells during differentiation and validation of 30 neural mRNA biomarkers for estimation of developmental neurotoxicity
Source: PLoS One. 2017 Dec 20;12(12):e0190066. doi: 10.1371/journal.pone.0190066 (PMC5738075; doi:10.1371/journal.pone.0190066)
Supplement: S3 Table — This function uses an iterative test of pairwise validation described by Vandesompele et al., 2002 [45]. Recommended coefficient variance should be <0.25 and M value should be <0.5 for homogenous samples. (PDF) [file pone.0190066.s007.pdf]

S3 Table. Target stability function analysis of the three reference genes using the Bio-Rad CFX manager 3.1 software system.

Target reference gene	Coefficient Variance	M Value
**Rplp1**	**0,1621**	**0,4092**
**Tbp**	**0,1243**	**0,3205**
**Hsp90ab1**	**0,1194**	**0,3184**

This function uses an iterative test of pairwise validation described by (Vandesompele et al., 2002). Recommended coefficient variance should be <0.25 and M value should be <0.5 for homogenous samples.
